# 

**Published:** 1888-11

**Authors:** 


					﻿, Vol. VIII.
NOVEMBER, 1888.
NO. 11.
JPHE
HOMEOPATHIC pH^lClAjl,
A Monthly Journal of Homoeopathic Materia Medica
and Clinical Medicine.
“He is a freeman whom truth makes free, and,all are slaves beside."
"Seek the Truth: come whence it may, cost what it will.”
Edited and published by
Edmund j. lee, ml id.,
AND
WALTER MI. JAMES, ML D.
PHILADELPHIA :
No. 11Q3 SPRUCE STREET.
* ;
TiOOSrliOTT:
ALFRED HEATH & CO., Sole Agents foe Great Britain,
114 Ebury Street, Eaton Square.
VS* Yearly Subscription, $2.50. invariably in advance. Single numbers, 25 cts.
Entered at the Philadelphia Poet-Offloe aa Second-Class Mall Matter.
				

